# Implantable Cardioverter-defibrillator Therapy for Syncope: An Educational Example of a Multicomponent Electrocardiographic Differential Diagnosis and the Application of Clinical Trial Data to an Individual Patient

**DOI:** 10.19102/icrm.2019.101002

**Published:** 2019-10-15

**Authors:** Daniel N. Pugliese, James A. Reiffel

**Affiliations:** ^1^Department of Medicine, Division of Cardiology, Columbia University Irving Medical Center, New York, NY, USA

**Keywords:** ICD, LV aneurysm, myocardial infarction, short QT, syncope

## Abstract

This is a report of a patient with a history of hypertension and myocardial infarction and a left ventricular ejection fraction of 35% who suffered a syncopal event. Her admitting electrocardiogram was compatible with her old myocardial infarction, an anteroseptal left ventricular aneurysm, left ventricular hypertrophy, and short-QT syndrome. The present report discusses how each of these might contribute individually and to some extent synergistically to producing syncope. She was treated with an implantable cardioverter-defibrillator (ICD), though she did not meet strict Multicenter Automatic Defibrillator Implantation Trial (MADIT), MADIT II, and Multicenter Unsustained Tachycardia Trial (MUSTT) patient characteristics. Her implant, however, was consistent with the 2014 Heart Rhythm Society/American College of Cardiology/American Heart Association consensus document regarding patients who do not match clinical trial enrollees but for whom ICD consideration is appropriate.

## Introduction

**Figure 1:**
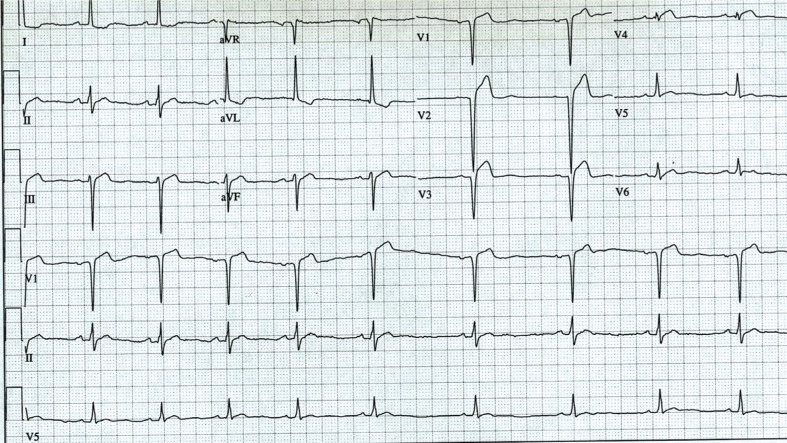
Twelve-lead ECG performed at the time of arrival to the hospital.

A 72-year-old female presented to the hospital emergency department for evaluation following an episode of abrupt unwitnessed syncope without prodromal symptoms. She was alone at home at the time of the incident. The syncopal episode lasted less than a minute before she awoke, with no focal neurological findings. Additionally, her clinical presentation was not consistent with vasovagal syncope. Her medical history was significant for an acute myocardial infarction (MI) five years prior and medication-controlled hypertension. Following her infarction, her left ventricular (LV) ejection fraction (LVEF) was approximately 35%, as determined by echocardiogram, and has remained stable on medical therapy since, including on a stress-echo study performed three months earlier, which was negative for ischemic changes other than her old infarcted, slightly dyskinetic region. She has had no subsequent anginal, congestive, or dysrhythmic symptoms and did not have any notable atrial or ventricular ectopy on ambulatory monitoring shortly after her infarction. She reported uncertainty regarding whether she sensed palpitations prior to her syncopal event or not. Her medications include aspirin, a β-blocker, an angiotensin-converting enzyme inhibitor, a diuretic (indapamide), and a statin. Upon arrival to the hospital, her vital signs were within normal limits. Additionally, the results of a screening neurological examination were unremarkable. The electrocardiogram (ECG) obtained at the time of arrival to the hospital is shown in **Figure 1**.

Specifically, **Figure 1** shows sinus rhythm with sinus arrhythmia (mean sinus cycle length: 992 ms). In this patient, the P–R interval was 190 ms, the QRS was 82 ms, and the mean QT and corrected QT intervals were both 310 ms. QS complexes are present in leads V1 to V3, consistent with her old anteroseptal infarction. Additionally, ST-segment elevation was noted in V1 to V4. The QRS complex and ST–T-wave patterns were unchanged from prior ECGs (not shown) subsequent to her infarction, and no ST depressions reciprocal to the ST elevations were present. Her short QT was observed on prior tracings but not addressed. Additionally, there was evidence of LV hypertrophy (LVH) based on voltage criteria in leads I and aVL, with associated repolarization abnormalities. Small but unremarkable U-waves were also observed to be present.

Upon reviewing the patient’s ECG, both the cardiologist and electrophysiology consult felt that the placement of an implantable cardioverter-defibrillator (ICD) was appropriate to recommend without any further testing being considered essential. In light of this patient’s multiple ECG findings, we sought to examine the mechanistic contributors suggested by the patient’s history and ECG and to consider whether the recommendation for an ICD without further testing in this case was truly reasonable.

## Discussion

The above ECG findings by themselves are compatible with several contributory causes for syncope. The most likely explanation is post-MI coronary artery disease with an LV aneurysm–associated ventricular tachyarrhythmia (VT). However, two less likely possible causes for or contributors to this patient’s syncope, based just on the ECG findings, also exist: arrhythmias associated with (1) LVH and/or (2) short-QT syndrome (SQTS).

### Coronary artery disease

When novel, ST elevation and Q-waves in the anterior precordial leads would suggest an acute/recent coronary syndrome. However, in the chronic setting, the persistent ST elevation in leads V1 to V4 accompanied by anteroseptal Q-waves, especially with no reciprocal ST depressions, suggests the presence of an LV aneurysm at the site of this patient’s prior infarct.^1–3^ This is compatible with her echocardiographic findings. When an LV aneurysm is associated with syncope, it is usually due to a VT. Multiple electrophysiological alterations that can contribute to VTs are present in and around the area of a prior MI (even more so in association with a ventricular aneurysm).^4–11^ They include (1) scar-related heterogeneity in conduction, action potential voltages, repolarization, and refractoriness that can produce reentry; (2) early and delayed afterdepolarizations; (3) alterations and increased responsiveness in mechanosensitive ion channel function, resulting in electrical instability; and (4) more.^4–11^ An accompanying awareness of palpitations prior to syncope may support the presence of a tachyarrhythmia, but this patient’s event was abrupt, without prodromal symptoms. Less commonly, LV aneurysms can result in syncope via cardioembolism from an LV thrombus.^12^ The absence of any prodromal or postsyncopal focal neurologic findings strongly suggests that a cerebral embolic event was not the cause.

Sustained VTs post-MI are an indication for ICD placement. When the patient has already had a documented VT event or has been resuscitated after cardiac arrest, the ICD is considered as secondary prevention.^13^ In contrast, primary-prevention ICD implantation is recommended in patients highly likely to suffer a first (and recurrent) VT event.^13,14^ Clinical trial results in the post-MI setting support primary ICD implantation in patients with an LVEF of 35% or less and nonsustained VT (NSVT) (based upon the Multicenter Automatic Defibrillator Implantation Trial; MADIT),^15^ patients with an LVEF of 30% or less without a need for NSVT (based on the MADIT II trial),^16^ or patients with an LVEF of 40% or less with NSVT plus inducible VT during electrophysiologic study (EPS) (based on the Multicenter Unsustained Tachycardia Trial; MUSTT).^17^ One might also consider the Sudden Cardiac Death in Heart Failure Trial (SCD-HeFT)^18^ as support for the decision. However, while this patient did have an LVEF of 35% or less as was required in SCD-HeFT, she did not have congestive symptoms, which were part of the criteria for enrollment in the SCD-HeFT trial, making SCD-HeFT not fully relevant to her case.

Notably, upon her presentation to the emergency room, this patient also did not strictly meet MADIT, MADIT II, or MUSTT trial criteria. She had not yet undergone ambulatory monitoring, so no information as to the presence or absence of NSVT was known. She did not have an LVEF of 30% or less and did not undergo electrophysiologic testing. Nonetheless, ICD implantation was unanimously recommended and pursued. This was consequent to not only the high suspicion of VT as a cause of her syncope in the setting of a prior MI, which may have been sufficient reason enough clinically, but also to the additional and possible contributory conditions noted on her ECG (ie, LVH and short QT interval).

### Left ventricular hypertrophy

LVH may contribute to a predilection for ventricular arrhythmias via multiple mechanisms, including reentrant arrhythmias in association with heterogeneous areas of fibrosis, inhomogeneity of repolarization and dispersion of refractoriness, and impaired cell-to-cell coupling; phase III–related arrhythmias due to hypertrophy-associated alterations in potassium, calcium, and late sodium repolarization currents; and/or subendocardial ischemia.^19–29^ LVH can also increase the development of atrial fibrillation, with a potential for thromboembolism or altered hemodynamics, but these were not present in this particular patient. Perhaps most relevant to this case, the risk for VT in the presence of LVH is increased (by as much as almost twofold) when coronary ischemic disease coexists,^30–32^ as was true in this woman. However, the ECG criteria for LVH in this patient were not marked, were not specific for any one single cause of LVH, and did not suggest hypertrophy severe enough to cause her syncopal event on its own. Moreover, LVH is common in patients with hypertension,^33^ whereas syncopal VT in otherwise uncomplicated hypertension is not. Further diagnostic evaluation (ie, echocardiography or other imaging modalities) would be needed for the differentiation of LVH etiologies. Hypertrophic cardiomyopathy and aortic valve disease were not present in this patient who on echocardiography had only mild concentric hypertrophy, which was likely related to her hypertension history. No thrombus was noted in either her left atrium or her aneurysm.

### Short-QT syndrome

The presence of a short QT interval can also predispose to VTs, and the extremely short duration of this patient’s QT interval in itself is compatible with those reported for SQTS.^34–36^ Notably, acquired causes of QT shortening such as metabolic derangements (eg, hyperkalemia, hypercalcemia, acidosis, hyperthermia), drugs (eg, digitalis), excess catecholamines or vagal tone, or acute myocardial ischemia were not present. While a diagnosis of SQTS can be considered in any individual in whom the corrected QT interval is less than 340 ms to 370 ms, the probability of this diagnosis increases as the QT duration decreases. This woman’s corrected QT interval of 310 ms is therefore striking.

Although the number of cases of SQTS reported thus far have been small, some criteria to recognize the syndrome have been proposed in a few reports to date.^37–40^ Perhaps the most widely cited are those of Gollub et al.,^37^ which consider not only the numerical value of the QT interval but also the clinical history, family history, and T-wave morphology. The latter consideration includes a short-J-point–T-peak interval, taller and more peaked T-waves (commonly in the septal leads),^37,38^ and the absence of an ST segment. Based on the above, this patient would have an “intermediate probability” of SQTS according to the Gollub criteria.^37^ However, while Gollub et al. says that SQTS may only be diagnosed when “the clinical story events occur in the absence of another identifiable etiology, including structural heart disease,” this may not be universally correct and certain additional caveats or examples should be added here. First, in the Gollub et al. review, the J-point–T-peak measurements were derived almost exclusively from males and the absolute numerical range they state may not apply to women. Second, given the small number of SQTS patients reported so far, the fact that SQTS may be caused by more than one specific gene abnormality and the fact that, in the long-QT syndrome, different T-wave patterns have been associated with different specific gene defects, we cannot be secure in a belief that all T-wave changes are likely to be uniform once the number of patients with each of the specific gene abnormalities is large enough to more fully assess. Third and perhaps most relevant here, the literature does not tell us how to evaluate the T-wave morphologic characteristics of SQTS when there is a superimposition of underlying cardiac disorder(s) that themselves can alter T-waves. Relatedly, if a patient with a true genetic short QT interval with all of the Gollub et al. criteria for SQTS independently suffers a large MI and then experiences a VT syncopal event a significant time later, how could one be absolutely certain which underlying contributor(s) were causative? Fourth, such underlying structural disorders have not been noted to produce a QT interval value as short as that seen in this patient.

The identified genetic defects in SQTS so far involve several different ion channels, including potassium, calcium, and sodium channels and an anion exchanger, as well as a gene implicated in carnitine transport.^34–36^ Importantly, however, genetic testing was not performed in this patient because its yield has usually been less than 25% and the findings would not have altered the ICD decision. That is, although ICD placement in SQTS is considered appropriate for secondary prevention,^40^ device implantation was already decided upon for this patient. Finally, SQTS is a rare clinical entity^36^ with only around 200 cases reported so far, with most experiencing their first clinical episode of syncope or VT at a young age. Nonetheless, while there may be doubt as to whether this patient accurately represents the SQTS profile, her QT was quite short and cannot be said with certainty as having been noncontributory to her syncopal event, and one patient in the Gollub et al. series was 70 years old.

Lastly and of potential importance to the future management of this patient, if ICD discharges were to become frequent and antiarrhythmic drug therapy were to become a consideration, her complex comorbidities would again play a treatment-determinant role. Quinidine and its congener, hydroquinidine, have been used with success in reducing VT events in patients with SQTS^34,35^ and likely would be considered as a first agent to try. Propafenone has also been attempted with some success in the SQTS setting,^35^ but, in the post-MI setting, a class Ic drug is more likely than quinidine to carry a proarrhythmic potential.^41^ Class III antiarrhythmics could be tried, having carried less risk post-MI, and in theory might increase her QT interval (as could quinidine), but there is little data about their use in SQTS patients so far and they can increase ventricular proarrhythmia syndromes in some LVH models.

## Clinical course

In the present patient, all electrolytes and myocardial biomarker tests were normal, including serum calcium. Further, serial ECGs were stable, and no acquired causes of SQTS or a history of familial sudden death were present in this case. The patient underwent coronary angiography and ventriculography, which confirmed an old left anterior descending occlusion and infarction with an anterior wall LV aneurysm without other coronary artery lesions. Twenty-four-hour monitoring by telemetry revealed short runs of nonsustained, pleomorphic ventricular tachycardia, which could be a consequence of any of the three risk conditions noted above. With her LVEF being less than 40% and her having NSVT and prior MI, the patient could have undergone an EPS for inducible VT so as to meet the MUSTT criteria for ICD implant. However, she instead received an ICD for the prevention of sudden cardiac death without undergoing EPS, as her multiple underlying predispositions including prior MI with LV aneurysm, LVH, and short QT interval were felt to warrant it. In this respect, her ICD implant was justified not strictly by the post-MI clinical trials noted above but rather by the Heart Rhythm Society/American College of Cardiology/American Heart Association expert consensus statement on the use of ICD therapy in patients who are either not included or who are not well-represented in clinical trials,^42^ in which the following statement can be found: “Randomized clinical trials study the effects of a particular treatment on a carefully selected and relatively homogeneous group of patients who meet specific inclusion and exclusion criteria for a particular clinical trial. Consistent with this approach, the indications for ICD therapy developed in the various guideline statements are limited to the specific populations of patients who participated in these clinical trials. Although the resulting guidelines are of great value, clinicians are often asked to make decisions regarding ICD therapy in patient populations who were not included or who were poorly represented in prior clinical trials. For these patients, there are no specific indications for ICD therapy … documentation of the reasons for ICD implantation [is] essential for all patients but [is] even more critical for those patients who have not been represented in clinical trials because the potential survival benefit must be calculated by taking the additional risks of comorbid conditions into account.” This case exemplifies the latter.
